# Epiconus syndrome induced only in the erect standing position in a patient with L1 compression fracture: a representative case report

**DOI:** 10.1002/ccr3.1392

**Published:** 2018-02-23

**Authors:** Kenji Kubota, Koki Abe, Sumihisa Orita, Kazuhide Inage, Miyako Suzuki, Jun Sato, Kazuki Fujimoto, Yasuhiro Shiga, Hirohito Kanamoto, Masahiro Inoue, Hideyuki Kinoshita, Masaki Norimoto, Tomotaka Umimura, Kazuhisa Takahashi, Seiji Ohtori

**Affiliations:** ^1^ Department of Orthopaedic Surgery Graduate School of Medicine Chiba University Chiba Japan

**Keywords:** Epiconus syndrome, expandable cage, myelography, neurological pathology, thoracolumbar spine, vertebral body fracture

## Abstract

In daily practice, when CT or MRI does not show a pathological lesion in a patient with persistent abnormal neurological signs, it is important to obtain imaging studies of the spine in dynamic position.

## Introduction

Epiconus syndrome is generally caused by intracanal lesions in the L4 to S2 spinal myelomeres, with neurological symptoms such as flaccid or spastic paresis, sensory deficits of the lower limbs, decreased reflexes, and vesicorectal dysfunction 1, 2, 3. We report a rare case that presented with transient leg weakness only in erect standing position, with no obvious intracanal lesions.

## Case Presentation

**Figure 1:**
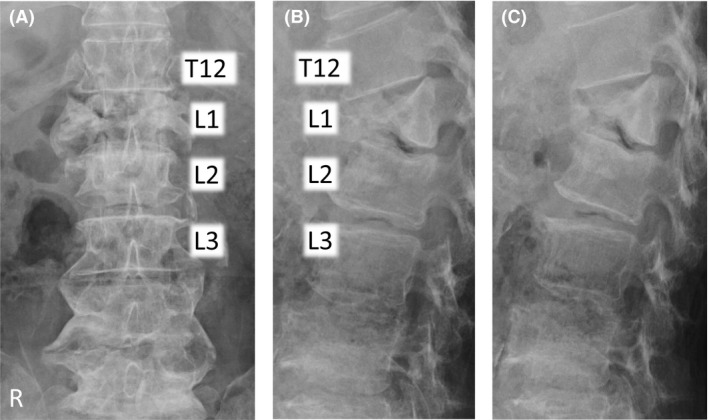
Plain radiographs in decubitus position. (A) Anteroposterior view, (B) lateral view in the flexion position, and (C) lateral view in the extension position on plain radiography performed on the examination table. These showed an L1 vertebral body fracture with an intact posterior wall. Flexion and extension views in the decubitus position showed no obvious malalignment of the vertebrae. Endplates of both sides were fractured without involvement of the posterior wall of the vertebral body; this was classified as AOS type A2.

**Figure 2:**
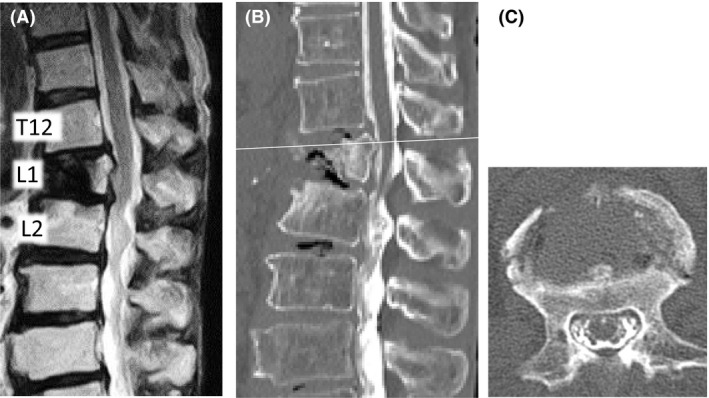
Magnetic resonance imaging and computed tomography after myelography. (A) T2‐weighted magnetic resonance imaging (MRI) of a sagittal section. (B) Computed tomography (CT) of a sagittal section after myelography in the supine position revealed that the termination of the spinal cord was at the upper level of the L2 vertebra. (C) This is shown at the white line on CT of an axial section. (B, C) The spinal canal with space around the cord behind the L1 vertebra.

**Figure 3:**
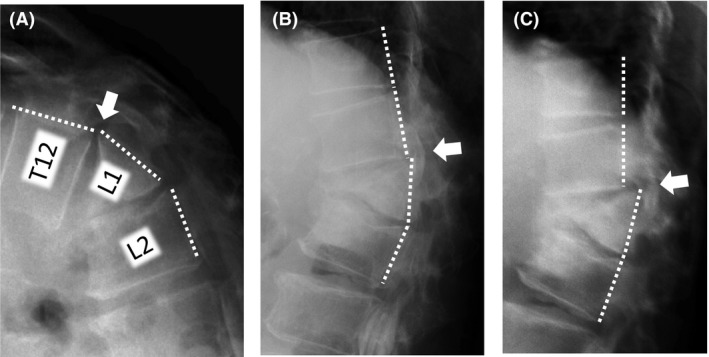
Changes in lateral myelogram views in different positions. Myelogram: (A) Lateral view in flexion position. (B) Lateral view in extension position while lying on the examination table. (C) Lateral view in erect standing position. The dotted white lines show the posterior edge of the vertebrae, and the white arrows indicate the gap of alignment between T12 and L1 in each position. The myelogram depicts the L1 vertebral body protruding into the spinal canal in (C).

**Figure 4:**
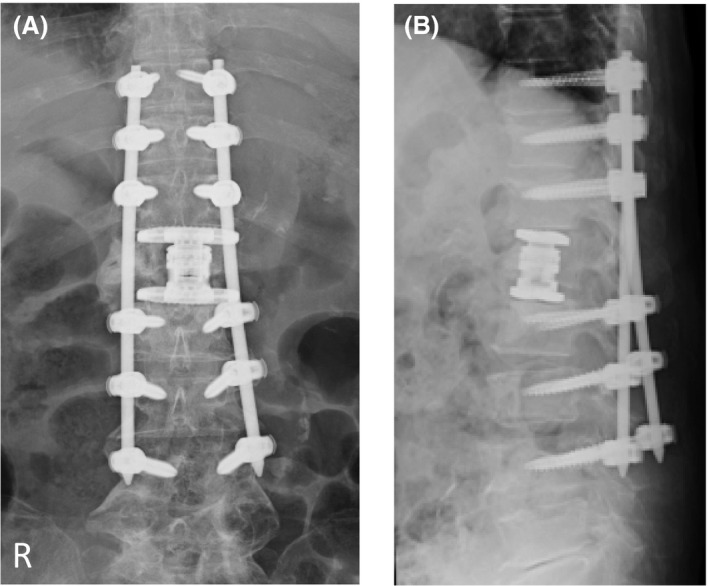
Plain postoperative radiographs. Results of postoperative examination with plain radiography: (A) anteroposterior view and (B) lateral view. The alignment of the posterior wall of the vertebrae prevented projection into the spinal canal. Rectangular endcaps were fitted between the inferior T12 and superior L2 endplate.

**Figure 5:**
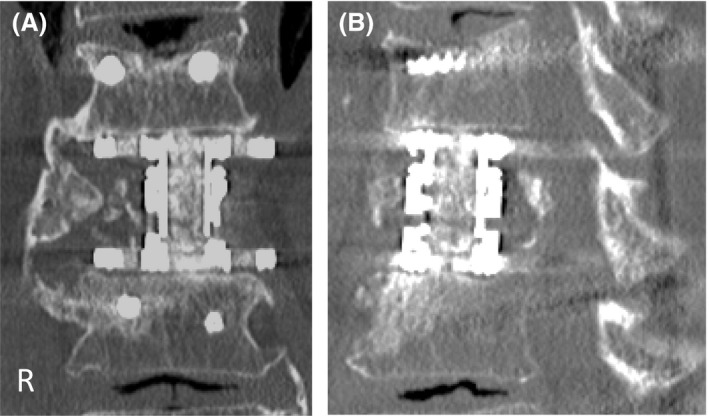
Computed tomography at 9 months after surgery. (A) Coronal and (B) sagittal sections of computed tomography images at 9 months after surgery. These showed continuity of trabecular bone between cage and endplate without implant failure or cage subsidence.

A 75‐year‐old woman presented with a 1‐year history of hyperkyphosis and gait disturbance. She often collapsed, with her knees sinking to the floor, but had no radicular pain in her legs if she stood upright. However, she was able to walk >500 m in a forward‐bending position with a walker. She had no muscle weakness, no pathological reflexes, and normal deep tendon reflexes in supine position. We detected no muscle atrophy, no vesicorectal dysfunction, and no sensory disturbance, other than numbness in her bilateral toes. We were not able to perform a standing neurological examination, because she could not maintain an erect standing position. We observed that she suddenly and repeatedly lost the ability to keep her bilateral knee joints in extended position, but had no leg pain on straightening her back in standing position. Plain X‐ray in decubitus position revealed an L1 vertebral body fracture with a nonprotruded posterior wall, and flexion/extension views showed no obvious malalignment of the vertebrae (Fig. 1). Computed tomography (CT) and magnetic resonance imaging (MRI) showed no apparent spinal stenosis (Fig. 2), but revealed termination of the spinal cord at the upper level of the L2 vertebra. However, only myelography in an erect standing position showed the L1 vertebral body protruding into the spinal canal (Fig. 3). The T12 vertebral body appeared as although it would slide down anteriorly on the “slope,” to push a triangular posterior L1 vertebral fragment into the canal. A prominent gap was observed in the anterior alignment of the spinal canal between the T12 and L1 vertebrae. Thus, we considered pathological compression of the epiconus caused by the L1 vertebral fragment in the erect standing position alone. This induced a lower neuron disorder at the distal lesion in the anterior horn of the spinal cord, which presented with transient bilateral knee hypotonia without severe leg pain, that is, a “transient epiconus syndrome.” The young adult mean scores on dual‐energy X‐ray absorptiometry of the lumbar spine and femoral neck were 76.0% and 73.1%, respectively. Although these values are not extreme, we thought that the L1 vertebral fracture was probably induced by osteoporosis. The patient had no history of trauma or malignant disease, and the L1 vertebra showed no evidence of tumor on MRI. Therefore, we performed combined anteroposterior subtotal corpectomy and reconstruction. After resecting the posterior fragment of the L1 vertebral body, we inserted a modular expandable cage (X‐Core 2, NuVasive, Inc. San Diego, CA, USA.) filled with autologous bone between the T12 and L2 vertebral bodies via the lateral approach. We then added posterior thoracolumbar spinal stabilization (T10–L4) using percutaneous pedicle screws (Fig. 4). The patient fully recovered independent gait in erect standing position with adequate bone union and no implant failure after surgery (Fig. 5).

## Discussion

**Figure 6:**
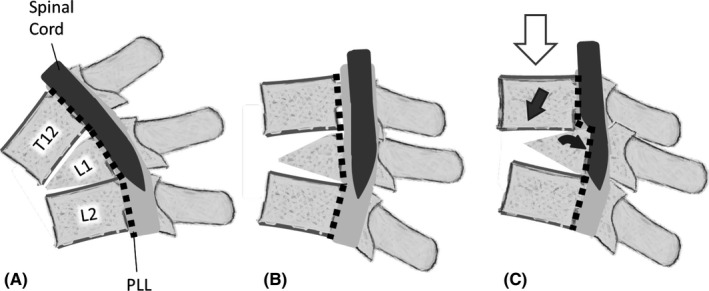
Schema of the positional relationship between T12 and L1 vertebrae. This figure illustrates the positional relationships between the T12–L2 vertebrae, the posterior longitudinal ligament, and the spinal cord in (A) flexion position, (B) extension position while lying on the examination table, and (C) erect standing position. The white arrow indicates gravitational force. The black arrow shows the direction of T12 downward slippage. The gray arrow shows the backward angulation and “relative retrolisthesis” of L1 into the spinal canal. PLL: Posterior longitudinal ligament.

The present case showed a unique, dynamic pathological epiconus syndrome that was only detected using myelography in erect standing position, and not with static modalities such as MRI and CT. Epiconus syndrome includes various lower motor neuron disorders in the anterior horn of the spinal cord caused by static compression of the L4–S2 myelomeres. Static intracanal lesions such as burst fractures and thoracic disk herniation are usually involved in the pathology 2, 3. The mechanism in the present case was considered to be the result of consecutive events, including subsidence of the fracture between the anterior and posterior fragments of the L1 vertebra, disruption of the intervertebral disk between T12 and L1, triangular posterior segment of the L1 vertebra, and a loosened posterior longitudinal ligament (PLL) (Fig. 6). We presume that the PLL was loosened by the vertical load in the erect standing position, allowing anterolisthesis of T12. At the same time, the posterior fragment of L1 was slightly angled backward by the vertical load of the T12 vertebra. Although no images clearly showed collapse of the T12/L1 facet joints, we surmise that these joints sustained spondylolisthesis. This series of movements led to “relative retrolisthesis” of L1 into the spinal canal. Compression at the T12‐L1 level of the spinal cord can reportedly cause sensory disturbance in the sole, foot, and around the anus, as well as muscle weakness 2. However, in the present case, we raise the possibility that transient compression on the anterior spinal cord contributed to the unique pathological condition in which only anterior horn cells were reversibly damaged.

In addition, the anatomical location of the epiconus is significant in the present case. The location of the epiconus is reported to range from the T10 vertebra to the T12–L1 intervertebral disk level and about 1.6 vertebrae proximal to the level of the termination of the spinal cord 3. The present case showed the conus at the upper level of the L2 vertebra, indicating the epiconus was located at about the T12/L1 level, just behind the L1 vertebral body.

A previous study showed that retropulsion of bony fragments into the spinal canal in patients with burst fractures resulted in a significant risk of neurological involvement in >35% of T11‐T12 fractures, >45% of L1 fractures, and >55% of fractures at L2 and below 4, indicating a high risk of a neurological disorder caused by L1 retrolisthesis in the present case.

In the present case, temporary compression of the ventral horn of the epiconus was thought to be caused by unusual retrolisthesis of a fragmented L1 fracture in erect standing position alone, inducing transient weakness in the limbs without significant vesicorectal dysfunction. Some cadaveric studies documented changes in the diameter of the spinal canal and intervertebral foramen in the flexion‐extension position 5, 6. Other studies reported that vertebral osteoporotic fractures induced delayed neurological disturbances through violation of the posterior cortex with retropulsion into the spinal canal, usually 1 week to 12 months after the injury 7, 8, 9. However, no report described transient paralysis caused by dynamic pathology as in the present case. Thus, a possible occult epiconus syndrome should be considered in patients with compression or burst vertebral fractures that can only be detected with dynamic assessment.

## Conclusions

We reported a rare case of epiconus syndrome resulting from compression due to dynamic instability of a vertebral segment. Transient protrusion of the L1 vertebra in an erect standing position was only identified using dynamic myelography.

## Authorship

KK: main author. KA: was responsible for writing and reviewing the manuscript. SO: performed critical revisions of the text. KI: was responsible for writing and reviewing the manuscript. MS and JS: were responsible for literature review. KF, YS, and HK: provided direct care to the patient. MI, HK, MN, and TU: were responsible for writing and reviewing the manuscript. KT and SO: coordinated and supervised the writing process.

## Conflicts of Interest

None declared.
